# Design, Synthesis, and Antifungal Activity of New *α*-Aminophosphonates

**DOI:** 10.1155/2011/678101

**Published:** 2011-09-26

**Authors:** Zahra Rezaei, Soghra Khabnadideh, Kamiar Zomorodian, Keyvan Pakshir, Setareh Nadali, Nadia Mohtashami, Ehsan Faghih Mirzaei

**Affiliations:** ^1^Department of Medicinal Chemistry and Pharmaceutical Sciences Research Center, Faculty of Pharmacy, Shiraz University of Medical Sciences, Shiraz 71345, Iran; ^2^Department of Parasitology, Faculty of Medicine, Shiraz University of Medical Sciences, Shiraz, Iran; ^3^Department of Medicinal Chemistry, Faculty of Pharmacy, Kerman University of Medical Sciences, Kerman, Iran

## Abstract

*α*-Aminophosphonates are bioisosteres of amino acids and have several pharmacological activities. These compounds have been synthesized by various routes from reaction between amine, aldehyde, and phosphite compounds. In order to synthesize *α*-aminophosphonates, catalytic effect of CuCl_2_ was compared with FeCl_3_. Also all designed structures as well as griseofulvin were docked into the active site of microtubule (1JFF), using Autodock program. The results showed that the reactions were carried out in the presence of CuCl_2_ in lower yields, and also the time of reaction was longer in comparison with FeCl_3_. The chemical structures of the new compounds were confirmed by spectral analyses. The compounds were investigated for antifungal activity against several fungi in comparison with griseofulvin. An indole-derived bis(*α*-aminophosphonates) with the best negative ΔG in docking study showed maximum antifungal activity against *Microsporum canis,* and other investigated compounds did not have a good antifungal activity.

## 1. Introduction

The *α*-aminophosphonates are amino acid analogues, which have found a wide range of applications in the areas of industrial, agricultural, and medicinal chemistry owing to their biological and physical properties as well as their utility as synthetic intermediates [1–5]. As a kind of natural amino acid analogues, *α*-aminophosphonates constitute an important class of compounds with diverse biological activities. The activity of *α*-aminophosphonates as pharmacogenic agents [6] is reported in the literature. Also it has been reported that some alkyl-substituted phosphonate compounds have antifungal activity [7, 8], antibacterial activity [9, 10], antitumor effects [11–13], and antiviral activity [14].

Three-component synthesis starting from aldehydes, amines and diethyl phosphite or triethyl phosphite have been reported by using Lewis and Bronsted acid catalysts such as LiClO4 [15], InCl_3_ [16], AlCl_3_ [17], lanthanide triflates/magnesium sulfate [18], SbCl_3_/Al_2_O_3_ [19], TaCl_5_-SiO_2_ [20], CF_3_CO_2_H [21], scandium (tris-dodecyl sulfate) [22], BF_3_·Et_2_O [23], M(OTf)_n _   [24], and M(ClO_4_)_n_ [25], though, many of these methods suffer from some drawbacks such as long reaction times, low yields of the products, requiring stoichiometric amounts of catalysts, costly and moisture sensitive catalysts, and use of highly toxic or toxic catalysts. More recently, ZrOCl_2_·8H_2_O [26] or ZrO(ClO_4_)_2_·6H_2_O [27] and TiO_2_ [28] are reported to be effective catalysts for the formation of *α*-aminophosphonates using a three- component system composing of aldehydes/ketones, amines, and diethylphosphite under neat conditions. Recently, we have reported one-pot three-component synthesis starting from aldehydes, amines and diethylphosphite using FeCl_3_ as a catalyst to formation of *α*-aminophosphonates [29]. As FeCl_3_ suffers from being hygroscopic and is also a corrosive material, in this study the catalyst effect of CuCl_2_ was compared with FeCl_3_ for *α*-aminophosphonates preparation.

As it has been reported that *α*-aminophosphonates have antifungal and cytotoxic activity [7, 8, 29], in this study a series of *α*-aminophosphonates was designed having aromatic aldehydes and amines with Cl and methoxy moiety similar to griseofulvin structure. Griseofulvin inhibits the growth of fungal cells by inducing abnormal mitosis. It has been reported that griseofulvin blocked the cells at G2/M phase of cell cycle and caused a significant depolymerisation of the spindle microtubules [30]. Because the griseofulvin binding site partially overlaps with the paclitaxel site in tubulin [30], therefore, microtubul complexed with paclitaxel (1JFF) was obtained from Protein Data Bank for docking studies. Autodock program used and all designed structures as well as griseofulvin were docked into the active site of 1JFF. In addition, we synthesized and investigated antifungal activity of some new *α*-aminophosphonates in comparison with griseofulvin.

## 2. Results and Discussion

### 2.1. Chemistry

**Scheme 1:**
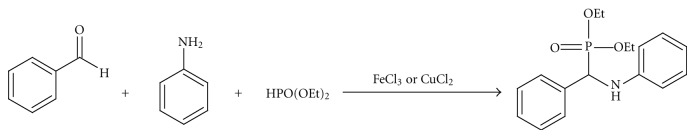
Three-component reaction of aromatic aldehydes with amine and diethylphosphite.

In order to synthesize *α*-aminophosphonates, the three components, aldehyde (benzaldehyde, 5.0 mmol), aromatic amine (aniline, 5.0 mmol), and diethyl phosphate (5.5 mmol), were reacted in the presence of catalytic amount (0.1 mmol) of FeCl_3_ or CuCl_2_ (Scheme 1). The reaction completely proceeded after 90 min with 73% yield in the presence of FeCl_3_, but the reaction did not completely proceed even after 24 h using CuCl_2_. The reactions were repeated with several aldehydes, amines, and diethyl phosphates with similar molar ratios as above in the presence of catalytic amount of FeCl_3_ or CuCl_2_. The reactions proceeded between 30–120 min in excellent isolated yields (73–84%) in the presence of FeCl_3_, but CuCl_2_ was not an effective catalyst like FeCl_3_. The results of this study are summarized in Table 1.

In this study 21 compounds were synthesized. The synthesis of compounds **1**, **8**, **11**, **12**, **14**, and **20** was carried out in the presence of catalytic amount of FeCl_3_ or CuCl_2_ (Table 1). The reactions proceed between 30–120 min in excellent isolated yields (73–84%) using FeCl_3_, but the reaction takes 24 h using CuCl_2_. However, it has been reported that metal chloride or metal halide are efficient catalyst for preparation of aminophosphonate by three-component reaction [31] but it seems that CuCl_2_ is not very efficient catalyst for formation of *α*-aminophosphonates in this condition. As our aim was comparison of the catalytic effect of CuCl_2_ with FeCl_3_ under same conditions, hence, other conditions were ignored. All compounds were synthesized by one-pot three-component synthesis using FeCl3 as a catalyst. The reactions completely proceeded after 30–180 min in excellent isolated yields (68–90%) in the presence of FeCl_3_ (Table 2). 

**Figure 1:**
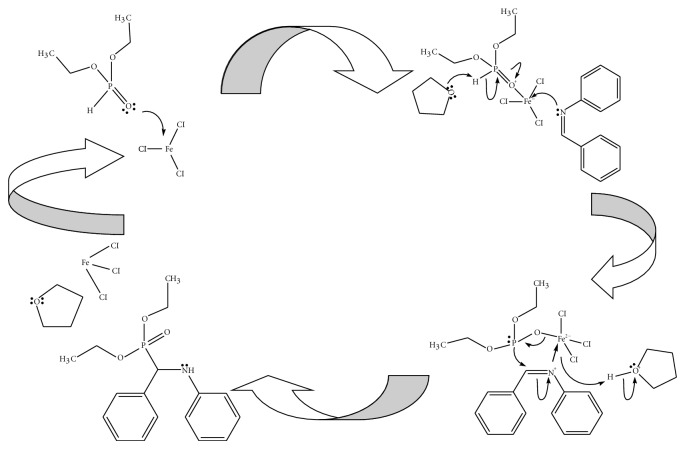
Proposed mechanism for catalytic effect of FeCl_3_.

The recommended mechanism for preparation of *α*-aminophosphonates using FeCl_3_ as a catalyst is shown in Figure 1. As shown in Figure 1, the reaction starts with activation of diethylphosphite a tautomer form in which the P (V) turns to P (III) with a free par of electron. Then the nitrogen of Schiff base that is formed in the first step of *α*-aminophosphonates formation donates a pair of electron to make a coordinante bond with FeCl_3_. This makes nitrogen positively charged which induces partial positive charge on sp^2^ carbon. The free pair of electrons of phosphorus attacks to the partially positively charged carbon and a cyclic current of electron displacement protonates nitrogen and detaches the FeCl_3_ to enter the new cycle. It seems that CuCl_2_ is not efficient as FeCl_3_ for attending to this mechanism for formation of *α*-aminophosphonates.

### 2.2. Modeling

**Figure 2:**
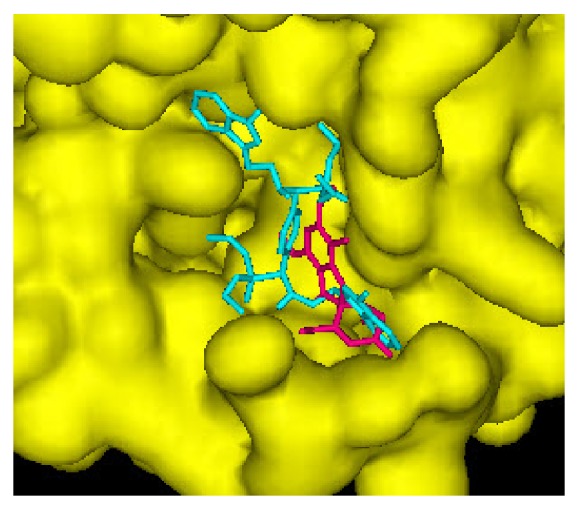
Accommodation of Griseofulvin (red) and compound **21 **(blue) in the active site of 1JFF.

All the compounds (Table 2) as well as griseofulvin were docked into the active site of microtubule, which was obtained from Protein Data Bank (1JFF) using Autodock 4.2. All synthesized compounds were characterized by a docking mode in the active site of the microtubule. Compound **21** showed cytotoxic activity in our previous study [29]. However, this compound has indole moiety like vinca alkaloids but binds to the paclitaxel site in 1JFF like griseofulvin (Figure 2). Therefore, antifungal activity of this compound was investigated in comparison with griseofulvin. According to obtained ΔG, compound **21** had the maximum negative ΔG and compound **15** had the lowest negative ΔG (Table 3); other compounds had ΔG close to griseofulvin. Although compound **21** with maximum negative ΔG had the best MIC but there was no correlation between antifungal activity and ΔG for other compounds.

### 2.3. Biological Assay

The synthesized compounds **1**–**21 **were evaluated for antifungal activity against *Candida albicans*,* Candida tropicalis*,* Aspergillus flavus*,* Microsporum canis*,* Microsporum gypseum*,* Trichophyton mentagrophyte*, and* Epidermophyton floccosum*. Agar dilution assay and microdilution method were used to establish the Minimum Inhibitory Concentration (MIC). The results are presented in Table 4. As shown in Table 4 compounds **1**,** 7**, and** 9** showed very low antifungal activity against *Trichophyton mentagrophytes*. Compound **1** also showed very low antifungal activity against *Microsporum gypseum*. Compound **21 **was the most active compound against *Microsporum canis*. This compound was previously evaluated in vitro for cytotoxicity effect and showed moderate cytotoxicity activity [29]; here this compound was evaluated for antifungal activity the MIC value found 5 *μ*g/mL, and the MIC for compound **21** was better than MIC for griseofulvin. Compound **21** is a bis-phosphonate, and it has an indole ring system, perhaps this moiety causes its antifungal activity. Also this compound had the better ΔG in docking study. Nevertheless, it has been reported that aminophosphonates have antifungal activity against phytopathogenic fungi [8, 14]; our synthesized compounds did not show antifungal activity against tested human pathogenic fungi. Song and coworkers reported that antifungal activity of aminophosphonates is related to stereochemistry of them [8]; therefore, may be the antifungal activity of our compound is related to stereochemistry of them. Therefore, we suggest that antifungal evaluation should be done for each enantiomer separately.

## 3. Experimental

All solvents and reagents were purchased from Sigma or Merck Chemical Companies. The products were purified by column chromatography techniques. NMR spectra were recorded on a Brucker Avance DPX 500 MHZ instrument. Mass spectra were recorded on a Hewlett-Packard GC-MS.

### 3.1. General Procedures for the Synthesis of Compounds

To a mixture of aldehyde (2 mmol), amine (1 mmol), and diethylphosphite (2.2 mmol) was added FeCl3 in THF (0.1 mmol) and stirred at 60°C for the appropriate reaction time. After completion of the reaction, EtOAc (10 mL) was added to the mixture. The mixture was washed with H_2_O (10 mL). The organic phase was separated and dried over anhydrous Na_2_SO_4_. The solvent was evaporated in vacuo, and the resulting crude material was purified by chromatography on a short column of silica gel (EtOAc/petroleum ether, 1/3) and then recrystallized from petroleum benzine/dichloromethane (4/1) to afford the pure *α*-aminophosphonates.

**Diethyl [Anilino(4-hydroxy-3-methoxyphenyl)methyl]phosphonate (1)**

This compound was synthesized after 120 min (84%. Mp = 95.4°C). ^1^H NMR (500 MHz, CDCl_3_): 7.15 (t, 2H, *J* = 6.6 Hz, ArH), 7.04 (s, 1H, ArH), 6.98 (d, 1H, *J* = 6.6 Hz, ArH), 6.90 (d, 1H, *J* = 8.3 Hz, ArH), 6.74 (t, 1H, *J* = 6.6 Hz, ArH), 6.64 (d, 2H, *J* = 6.6 Hz, ArH), 4.72 (d, 1H, *J*_CHPO_ = 21.6 Hz, CHP), 4.12–4.18 (m, 2H, OCH_2_CH_3_), 3.97–4.02 (m, s, 1H, OCH_2_CH_3_), 3.87 (s, 3H, OCH_3_), 3.72–3.77 (m, 1H, OCH_2_CH_3_), 1.32 (t, 3H, *J* = 8.3 Hz, CH_3_), 1.18 (t, 3H, *J* = 8.3 Hz, CH_3_); ^13^C NMR (125 MHz, CDCl_3_): 147.35 (Ar-C), 146.93 (Ar-C), 146.81 (Ar-C), 145.97 (Ar-C), 129.57 (Ar-C), 127.87 (Ar-C), 114.93 (Ar-C), 114.32 (Ar-C), 110.67 (Ar-C), 63.72 (d, *J*_PCH2_ = 7.5 Hz, OCH_2_CH_3_), 56.38 (d, *J*_PCH_ = 157.8 Hz, CHP), 55.64 (OCH_3_), 16.87 (d, *J*_PCH3_ = 5.0 Hz, CH_3_), 16.70 (d, *J*_PCH3_ = 5.0 Hz, CH_3_); MS: (m/z%), 363 (M^+^, 21.8), 228 (100), 137 (9.3).

**Diethyl [(4-Nitrophenyl amino) (3,4,5-trimethoxy) methyl]phosphonate (2)**

This compound was synthesized after 150 min (72%. Mp = 89.4°C). ^1^H NMR (500 MHz, CDCl_3_): 8.07 (d, 2H, *J* = 6.6 Hz, ArH), 6.96 (s, 2H, ArH), 6.64 (s, 2H, ArH), 5.72 (t, 1H, *J* = 7.1 Hz, NH), 4.75 (d, 1H, *J*_CHPO_ = 23.8 Hz, CHP), 4.12–4.21 (m, 1H, OCH_2_CH_3_), 3.98–4.03 (m, 1H, OCH_2_CH_3_), 3.85 (s, 9H, OCH_3_), 3.70–3.75 (m, 1H, OCH_2_CH_3_), 1.34 (t, 3H, *J* = 6.6 Hz, CH_3_), 1.18 (t, 3H, *J* = 6.6 Hz, CH_3_); ^13^C NMR (125 MHz, CDCl_3_): 154.06 (Ar-C), 152.21 (Ar-C), 136.66 (Ar-C), 130.45 (Ar-C), 126.53 (Ar-C), 112.88 (Ar-C), 105.12 (Ar-C), 64.03 (d, ^3^ *J*_PC_ = 6.3 Hz, OCH_2_CH_3_), 59.74 (d, ^3^ *J*_PC_ = 7.5 Hz, CH_3_), MS: (m/z%), 545 (M^+^, 5.7), 317 (100), 271 (5), 181 (5.7).

**Diethyl [(4-Hydroxy, 3-methoxy phenyl) (4-nitrophenyl amino) methyl]phosphonate (3)**

This compound was synthesized after 180 min (78%. Mp = 138.1°C). ^1^H NMR (500 MHz, CDCl_3_): 8.04 (d, 2H, *J* = 6.6 Hz, ArH), 7.00 (d, 1H, *J* = 6.6 Hz, ArH), 6.97 (d, 2H, *J* = 6.6 Hz, ArH), 6.95 (s, 1H, ArH), 6.64 (d, 2H, *J* = 6.6 Hz, ArH), 5.86 (t, 1H, *J* = 7.1 Hz, NH), 4.76 (d, 1H, *J*_CHPO_ = 23.3 Hz, CHP), 4.13–4.2 (m, 2H, OCH_2_CH_3_), 3.95–4.00 (m, 1H, OCH_2_CH_3_), 3.88 (s, 3H, OCH_3_), 3.67–3.76 (m, 1H, OCH_2_CH_3_), 1.32 (t, 3H, *J* = 6.6 Hz, CH_3_), 1.17 (t, 3H, *J* = 6.6 Hz, CH_3_); ^13^C NMR (125 MHz, CDCl_3_): 152.34 (Ar-C), 147.52 (Ar-C), 146.38 (Ar-C), 139.45 (Ar-C), 126.49 (Ar-C), 126.44 (Ar-C), 121.35 (Ar-C), 115.18 (Ar-C), 112.86 (Ar-C), 110.47 (Ar-C), 64.04 (d, ^3^ *J*_PC_ = 6.3 Hz, OCH_2_CH_3_), 56.38 (d, ^3^ *J*_PC_ = 7.5 Hz, CH_3_), 55.07 (OCH_3_)MS: (m/z%), 410 (M^+^, 5.5), 273 (100), 227 (8.8), 137 (7.7).

**Diethyl [(3,4-Dimethoxy phenyl amino) (3,4,5-trimethoxy phenyl) methyl]phosphonate (4)**

This compound was synthesized after 120 min (87%. Mp = 105°C). ^1^H NMR (500 MHz, CDCl_3_): 6.72 (s, 2H, ArH), 6.68 (d, 1H, *J* = 6.6 Hz, ArH), 6.31 (s, 1H, ArH), 6.11 (d, 1H, *J* = 8.3 Hz, ArH), 4.60 (d, 1H, *J*_CHPO_ = 23.3 Hz, CHP), 4.11–4.19 (m, 2H, OCH_2_CH_3_), 3.98–4.03 (m, 1H, OCH_2_CH_3_), 3.84 (s, 9H, OCH_3_), 3.81 (s, 6H, OCH_3_), 3.75–3.79 (m, 1H, OCH_2_CH_3_), 1.32 (t, 3H, *J* = 6.6 Hz, CH_3_), 1.18 (t, 3H, *J* = 6.6 Hz, CH_3_); ^13^C NMR (125 MHz, CDCl_3_): 153.77 (Ar-C), 150.23 (Ar-C), 142.66 (Ar-C), 141.55 (Ar-C), 138.04 (Ar-C), 132.17 (Ar-C), 113.25 (Ar-C), 105.21 (Ar-C), 100.5 (Ar-C), 63.65 (d, *J*_PC_ = 6.3 Hz, OCH_2_CH_3_), 57.10 (d, *J*_PC_ = 147.2 Hz CHP), 56.91 (OCH_3_), 56.58 (OCH_3_), 56.1 (OCH_3_), 16.88 (d, *J*_PCH_ = 5.7 Hz, CH_3_), 16.70 (d, *J*_PCH_ = 5.7 Hz, CH_3_); MS: (m/z%), 469 (M^+^, 7.1), 331 (100), 300 (3.5), 195 (11.9).

**Diethyl [(3,4-Dimethoxy phenyl) (4-nitrophenyl amino) methyl]phosphonate (5)**

This compound was synthesized after 180 min (80%. Mp = 99.2°C). ^1^H NMR (500 MHz, CDCl_3_): 8.04 (d, 2H, *J* = 6.6 Hz, ArH), 7.01 (d, 2H, *J* = 6.6 Hz, ArH), 6.86 (s, 2H, ArH), 6.62 (d, 2H, *J* = 6.6 Hz, ArH), 5.87 (t, 1H, NH), 4.74 (d, 1H, *J*_CHPO_ = 20.01 Hz, CHP), 4.13–4.18 (m, 2H, OCH_2_CH_3_), 3.94–3.98 (m, 1H, OCH_2_CH_3_), 3.69–3.74 (m, 1H, OCH_2_CH_3_), 1.32 (t, 3H, *J* = 6.6 Hz, CH_3_), 1.16 (t, 3H, *J* = 6.6 Hz, CH_3_); ^13^C NMR (125 MHz, CDCl_3_): 152.35 (Ar-C), 149.78 (Ar-C), 149.60 (Ar-C), 139.45 (Ar-C), 127.15 (Ar-C), 126.48 (Ar-C), 120.63 (Ar-C), 112.86 (Ar-C), 111.67 (Ar-C), 111.08 (Ar-C), 63.98 (d, *J*_PC_ = 7.5 Hz, OCH_2_CH_3_), 56.32 (d, *J*_PC_ = 149.2 Hz CHP), 55.03 (OCH_3_), 56.58 (OCH_3_), 16.85 (d, *J*_PCH_ = 7.5 Hz, CH_3_), 16.70 (d, *J*_PCH_ = 7.5 Hz, CH_3_); MS: (m/z%), 424 (M^+^, 5.5), 287 (100), 241 (7), 149 (2.9).

**Diethyl [(5-Chloro-2-methylphenyl amino) (3,4,5-trimethoxy phenyl) methyl]phosphonate (6)**

This compound was synthesized after 120 min (76%. Mp = 107°C). ^1^H NMR (500 MHz, CDCl_3_):6.96 (s, 1H, ArH), 6.94 (d, 1H, *J* = 8.3 Hz, ArH), 6.7 (s, 2H, ArH), 6.45 (s, 1H, ArH), 4.67 (d, 1H, *J*_CHPO_ = 26.60 Hz, CHP), 4.09–4.17 (m, 2H, OCH_2_CH_3_), 3.96–4.01 (m, 1H, OCH_2_CH_3_), 3.85 (s, 9H, OCH_3_), 3.72–3.77 (m, 1H, OCH_2_CH_3_), 2.24 (s, 3H, CH_3_), 1.29 (t, 3H, *J* = 6.6 Hz, CH_3_), 1.16 (t, 3H, *J* = 6.6 Hz, CH_3_); ^13^C NMR (125 MHz, CDCl_3_): 153.86 (Ar-C), 145.98 (Ar-C), 138.28 (Ar-C), 132.85 (Ar-C), 131.41 (Ar-C), 121.55 (Ar-C), 118.32 (Ar-C), 111.78 (Ar-C), 105.14 (Ar-C), 63.76 (d, *J*_PC_ = 6.3 Hz, OCH_2_CH_3_), 57.30 (d, *J*_PC_ = 147.2 Hz CHP), 56.60 (OCH_3_), 17.47 (CH3), 16.84 (d, *J*_PCH_ = 7.5 Hz, CH_3_), 16.66 (d, *J*_PCH_ = 7.5 Hz, CH_3_); MS: (m/z%), 457 (M^+^, 5.3), 320 (100), 181 (6.6).

**Diethyl [(5-Chloro-2-methylphenyl amino) (4-methoxy phenyl) methyl]phosphonate (7)**

This compound was synthesized after 120 min (77%. Mp = 102.4°C). ^1^H NMR (500 MHz, CDCl_3_): 7.40 (d, 2H, *J* = 6.6 Hz, ArH), 6.97 (s, 1H, ArH), 6.92 (d, 2H, *J* = 6.6 Hz, ArH), 6.64 (d, 1H, *J* = 6.6 Hz, ArH), 6.43 (d, 1H, *J* = 6.6 Hz, ArH), 4.71 (d, 1H, *J*_CHPO_ = 23.30 Hz, CHP), 4.11–4.19 (m, 2H, OCH_2_CH_3_), 3.95–4.00 (m, 1H, OCH_2_CH_3_), 3.82 (s, 3H, OCH_3_), 3.71–3.76 (m, 1H, OCH_2_CH_3_), 2.25 (s, 3H, CH_3_), 1.32 (t, 3H, *J* = 6.6 Hz, CH_3_), 1.17 (t, 3H, *J* = 6.6 Hz, CH_3_); ^13^C NMR (125 MHz, CDCl_3_): 159.88 (Ar-C), 145.85 (Ar-C), 132.83 (Ar-C), 131.35 (Ar-C), 129.19 (Ar-C), 127.54 (Ar-C), 121.59 (Ar-C), 118.10 (Ar-C), 114.63 (Ar-C), 111.71 (Ar-C), 63.68 (d, *J*_PC_ = 3.75 Hz, OCH_2_CH_3_), 56.28 (d, *J*_PC_ = 152 Hz CHP), 55.66 (OCH_3_), 17.48 (CH3), 16.86 (d, *J*_PCH_ = 5.75 Hz, CH_3_), 16.68 (d, *J*_PCH_ = 5.75 Hz, CH_3_); MS: (m/z%), 397 (M^+^, 7.0), 260 (100), 121 (17.8).

**Diethyl [(3,4-Dimethoxyphenyl) (phenyl amino) methyl]phosphonate (8)**

This compound was synthesized after 120 min (82%. Mp = 103°C). ^1^H NMR (500 MHz, CDCl_3_): 7.13 (t, 2H, *J* = 8.3 Hz, ArH), 7.04 (d, 2H, *J* = 8.3 Hz, ArH), 6.85 (s, 1H, ArH), 6.72 (t, 1H, *J* = 8.3 Hz, ArH), 6.63 (d, 2H, *J* = 8.3 Hz, ArH), 4.72 (d, 1H, *J*_CHPO_ = 18.30 Hz, CHP), 4.10–4.17 (m, 2H, OCH_2_CH_3_), 3.96–4.01 (m, 1H, OCH_2_CH_3_), 3.88 (s, 6H, OCH_3_), 3.71–3.76 (m, 1H, OCH_2_CH_3_), 1.31 (t, 3H, *J* = 8.3 Hz, CH_3_), 1.17 (t, 3H, *J* = 8.3 Hz, CH_3_); ^13^C NMR (125 MHz, CDCl_3_): 149.52 (Ar-C), 149.14 (Ar-C), 146.87 (Ar-C), 129.56 (Ar-C), 128.65 (Ar-C), 120.63 (Ar-C), 118.84 (Ar-C), 114.31 (Ar-C), 111.54 (Ar-C), 111.25 (Ar-C), 63.64 (d, *J*_PC_ = 4.60 Hz, OCH_2_CH_3_), 56.83 (d, *J*_PC_ = 187.25 Hz CHP), 56.30 (OCH_3_), 16.88 (d, *J*_PCH_ = 5.80 Hz, CH_3_), 16.72 (d, *J*_PCH_ = 5.80 Hz, CH_3_); MS: (m/z%), 379 (M^+^, 5.0), 242 (100), 151 (17.8).

**Diethyl [(4-Chloro-2-nitrophenyl amino) (4-hydroxy-3-methoxyphenyl) methyl]phosphonate (9)**

This compound was synthesized after 180 min (70%. Mp = 187.4°C). ^1^H NMR (500 MHz, CDCl_3_): 8.22 (s, 1H, ArH), 7.30 (d, 1H, *J* = 6.6 Hz, ArH), 6.99 (d, 1H, *J* = 6.6 Hz, ArH), 6.93 (d, 2H, *J* = 6.6 Hz, ArH), 6.71 (s, 1H, ArH), 4.82 (d, 1H, *J*_CHPO_ = 23.8 Hz, CHP), 4.03–4.14 (m, 2H, OCH_2_CH_3_), 3.96–3.99 (m, 1H, OCH_2_CH_3_), 3.91 (s, 3H, OCH_3_), 3.71–3.76 (m, 1H, OCH_2_CH_3_), 1.28 (t, 3H, *J* = 6.6 Hz, CH_3_), 1.17 (t, 3H, *J* = 6.6 Hz, CH_3_); ^13^C NMR (125 MHz, CDCl_3_): 147.53 (Ar-C), 146.42 (Ar-C), 143.15 (Ar-C), 136.59 (Ar-C), 133.52 (Ar-C), 126.41 (Ar-C), 126.03 (Ar-C), 122.01 (Ar-C), 121.05 (Ar-C), 116.66 (Ar-C), 115.14 (Ar-C), 110.21 (Ar-C), 64.11 (d, *J*_PC_ = 8.0 Hz, OCH_2_CH_3_), 56.46 (d, *J*_PC_ = 150.9 Hz CHP), 55.23 (OCH_3_), 16.84 (d, *J*_PCH_ = 5.9 Hz, CH_3_), 16.75 (d, *J*_PCH_ = 5.9 Hz, CH_3_); MS: (m/z%), 444 (M^+^, 1.0), 307 (100), 290 (23.5), 273 (41.1), 151 (26.4).

**Diethyl [(3,4-Dimethoxy phenyl amino) (4-hydroxy-3-methoxy phenyl) methyl]phosphonate (10)**

This compound was synthesized after 120 min (81%. Mp = 103.2°C). ^1^H NMR (500 MHz, CDCl_3_): 7.00 (s, 2H, ArH), 6.81 (d, 1H, *J* = 6.6 Hz, ArH), 6.64 (d, 1H, *J* = 6.6 Hz, ArH), 6.26 (d, 1H, *J* = 6.6 Hz, ArH), 6.09 (d, 1H, *J* = 6.6 Hz, ArH), 4.60 (d, 1H, *J*_CHPO_ = 26.0 Hz, CHP), 4.07–4.16 (m, 2H, OCH_2_CH_3_), 3.93–3.98 (m, 1H, OCH_2_CH_3_), 3.85 (s, 6H, OCH_3_), 3.75 (s, 3H, OCH_3_), 3.69–3.73 (m, 1H, OCH_2_CH_3_), 1.29 (t, 3H, *J* = 8.3 Hz, CH_3_), 1.14 (t, 3H, *J* = 8.3 Hz, CH_3_); ^13^C NMR (125 MHz, CDCl_3_): 150.20 (Ar-C), 149.50 (Ar-C), 142.53 (Ar-C), 141.61 (Ar-C), 141.48 (Ar-C), 128.82 (Ar-C), 120.61 (Ar-C), 113.27 (Ar-C), 111.50 (Ar-C), 111.26 (Ar-C), 105.19 (Ar-C), 100.52(Ar-C), 63.60 (d, *J*_PC_ = 6.3 Hz, OCH_2_CH_3_), 56.90 (d,  *J*_PC_ = 147.2 Hz CHP), 56.32 (OCH_3_), 56.05 (OCH_3_), 16.86 (d, *J*_PCH_ = 5.7 Hz, CH_3_), 16.70 (d, *J*_PCH_ = 5.7 Hz, CH_3_); MS: (m/z%), 425 (M^+^, 25), 288 (100), 272 (3.5), 1490 (8.3).

**Diethyl [(Phenyl amino) (3,4,5-trimethoxy phenyl) methyl]phosphonate (11)**

This compound was synthesized after 30 min (81%. Mp = 109°C). ^1^H NMR (500 MHz, CDCl_3_): 7.16 (t, 2H, *J* = 7.25 Hz, ArH), 6.75 (t, 3H, *J* = 7.3 ArH), 6.65 (d, 2H, *J* = 7.56 Hz, ArH), 4.80 (d, 1H, *J*_CHPO_ = 41.7 Hz, CHP), 4.10–4.17 (m, 2H, OCH_2_CH_3_), 3.99–4.03 (m, 1H, OCH_2_CH_3_), 3.85 (s, 9H, OCH_3_), 3.75–3.79 (m, 1H, OCH_2_CH_3_), 1.32 (t, 3H, *J* = 7.3 Hz, CH_3_), 1.18 (t, 3H, *J* = 7.3 Hz, CH_3_); ^13^C NMR (125 MHz, CDCl_3_): 153.76 (Ar-C), 146.92 (Ar-C), 131.96 (Ar-C), 129.61 (Ar-C), 118.96 (Ar-C), 114.27 (Ar-C), 105.23 (Ar-C), 63.72 (d, *J*_PC_ = 5.1 Hz, OCH_2_CH_3_), 56.81 (d, *J*_PC_ = 149 Hz CHP), 56.57 (OCH_3_), 56.21 (OCH_3_), 16.87 (d, *J*_PCH_ = 5.6 Hz, CH_3_), 16.70 (d, *J*_PCH_ = 6.0 Hz, CH_3_); MS: (m/z%), 409 (M^+^, 7.4), 272 (100), 181 (5.4).

**Diethyl [4-Methoxyphenyl)(phenyl amino)methyl]phosphonate (12)**

This compound was synthesized after 60 min (76%. Mp = 102°C). ^1^H NMR (500 MHz, CDCl_3_): 7.42 (d, 2H, *J* = 7.5 Hz, ArH), 7.13 (t, 2H, *J* = 7.5 Hz, ArH), 6.90 (d, 2H *J* = 8.5 Hz, ArH), 6.72 (d, 2H, *J* = 8.5 Hz, ArH), 6.72 (t, 1H, *J* = 7.25 Hz, ArH), 6.63 (d, 2H, *J* = 8.2 Hz, ArH), 4.78 (s, 1H, NH), 4.76 (d, 1H, *J*_CHPO_ = 40.1 Hz, CHP), 4.11–4.17 (m, 2H, OCH_2_CH_3_), 3.96–4.0 (m, 1H, OCH_2_CH_3_), 3.80 (s, 3H, OCH_3_), 3.71–3.76 (m, 1H, OCH_2_CH_3_), 1.32 (t, 3H, *J* = 7.3 Hz, CH_3_), 1.17 (t, 3H, *J* = 7.3 Hz, CH_3_); ^13^C NMR (125 MHz, CDCl_3_): 159.74 (Ar-C), 146.79 (Ar-C), 129.57 (Ar-C), 129.38 (Ar-C), 128.11 (Ar-C), 118.76 (Ar-C), 114.47 (Ar-C), 114.31 (Ar-C), 63.62 (d, ^3^ *J*_PC_ = 4.75 Hz, OCH_2_CH_3_), 55.87 (d, ^3^ *J*_PC_ = 151 Hz, CHP), 55.64 (OCH_3_), 16.85 (d, *J*_PCH3_ = 5.7 Hz, CH_3_), 16.68 (d, *J*_PCH3_ = 5.7 Hz, CH_3_); MS: (m/z%), 349 (M^+^, 3.9), 212 (100), 121 (3.9).

**Diethyl [(4-Chloro-2-nitrophenyl amino) (3,4,5-trimethoxy phenyl) methyl]phosphonate (13)**

This compound was synthesized after 90 min (75%. Mp = 137°C). ^1^H NMR (500 MHz, CDCl_3_): 8.84 (d, 1H, *J* = 11.0 Hz, ArH), 8.19 (s, 1H, ArH), 6.67–677 (m, 3H, ArH), 4.79 (d, 1H, *J*_CHPO_ = 23.25 Hz, CHP), 4.09–4.14 (m, 2H, OCH_2_CH_3_), 4.03–4.07 (m, 1H, OCH_2_CH_3_), 3.97-3.99 (m, 1H, OCH_2_CH_3_), 3.84 (s, 9H, OCH_3_), 1.31-1.25 (m, 6H, *J* = 7.3 Hz, CH_3_); ^13^C NMR (125 MHz, CDCl_3_): 154.6 (Ar-C), 143.17 (Ar-C), 143.06 (Ar-C), 138.53 (Ar-C), 136.66 (Ar-C), 130.09 (Ar-C), 130.06 (Ar-C), 126.38 (Ar-C), 122.15 (Ar-C), 116.67 (Ar-C), 64.19 (d, *J*_PC_ = 7.1 Hz, OCH_2_CH_3_), 64.02 (d, *J*_PC_ = 7.25 Hz, OCH_2_CH_3_), 56.66 (OCH_3_), 56.32 (d, *J*_PC_ = 151 Hz CHP), 16.82 (d, *J*_PCH_ = 5.7 Hz, CH_3_), 16.73 (d, *J*_PCH_ = 5.70 Hz, CH_3_); MS: (m/z%), 488 (M^+^, 4.3), 351 (100), 195 (8.7).

**Diethyl [(Phenyl amino)(phenyl)methyl]phosphonate (14)**

This compound was synthesized after 90 min (73%. Mp = 77°C). ^1^H NMR (500 MHz, CDCl_3_): 7.53 (s, 2H, ArH), 7.38 (t, 2H, *J* = 7.5 Hz, ArH), 7.29-7.32 (m, 1H, ArH), 7.15 (t, 2H, *J* = 8.2 Hz, ArH), 6.74 (t, 1H, *J* = 7.5 Hz, ArH), 6.65 (d, 2H, *J* = 8.5 Hz, ArH), 4.82 (d, 1H, *J*_CHPO_ = 24.57 Hz, CHP), 4.11–4.21 (m, 2H, OCH_2_CH_3_), 3.96–4.01 (m, 1H, OCH_2_CH_3_), 3.70–3.75 (m, 1H, OCH_2_CH_3_), 1.33 (t, 3H, *J* = 7.0 Hz, CH_3_), 1.16 (t, 3H, *J* = 7.3 Hz, CH_3_); ^13^C NMR (125 MHz, CDCl_3_): 146.80 (Ar-C), 136.35 (Ar-C), 129.60 (Ar-C), 129.02 (Ar-C), 128.34 (Ar-C), 128.30 (Ar-C), 118.82 (Ar-C), 114.29 (Ar-C), 63.72 (d, *J*_PC_ = 4.8 Hz, OCH_2_CH_3_), 63.66 (d, *J*_PC_ = 4.8 Hz, OCH_2_CH_3_), 56.50 (d, *J*_PC_ = 149 Hz, CHP), 16.88 (d, *J*_PCH3_ = 5.7 Hz, CH_3_), 16.64 (d, *J*_PCH3_ = 5.8 Hz, CH_3_); MS: (m/z%), 319 (M^+^, 16), 182 (100).

**Diethyl [(3,4-Dimethoxy phenyl amino) (3,4-dimethoxy phenyl) methyl]phosphonate (15)**

This compound was synthesized after 45 min (75%. Mp = 115°C). ^1^H NMR (500 MHz, CDCl_3_): 6.99–7.01 (m, 2H, ArH), 6.82 (d, 1H, *J* = 8.1 Hz, ArH), 6.63 (d, 1H, *J* = 8.5 Hz, ArH), 6.29 (s, 1H, ArH), 6.08 (d, 1H, *J* = 7.1 Hz, ArH), 4.65 (s, 1H, NH), 4.59 (d, 1H, *J*_CHPO_ = 23.3 Hz, CHP), 4.07–4.16 (m, 2H, OCH_2_CH_3_), 3.93–3.98 (m, 1H, OCH_2_CH_3_), 3.85 (s, 6H, OCH_3_), 3.74 (s, 6H, OCH_3_), 3.69–3.74 (m, 1H, OCH_2_CH_3_), 1.29 (t, 3H, *J* = 6.6 Hz, CH_3_), 1.14 (t, 3H, *J* = 6.6 Hz, CH_3_); ^13^C NMR (125 MHz, CDCl_3_): 150.20 (Ar-C), 149.50 (Ar-C), 149.10 (Ar-C), 142.53 (Ar-C), 141.55 (Ar-C), 128.82 (Ar-C), 120.63 (Ar-C), 113.27 (Ar-C), 111.50 (Ar-C), 111.26 (Ar-C), 105.19 (Ar-C), 100.52 (Ar-C), 63.63 (d, *J*_PC_ = 3.0 Hz, OCH_2_CH_3_), 63.58 (d, *J*_PC_ = 3.0 Hz, OCH_2_CH_3_), 57.03 (d, *J*_PC_ = 151.0 Hz CHP), 56.99 (OCH_3_), 56.32 (OCH_3_), 56.23 (OCH_3_), 56.05 (OCH_3_), 16.86 (d, *J*_PCH_ = 5.6 Hz, CH_3_), 16.70 (d, *J*_PCH_ = 5.6 Hz, CH_3_); MS: (m/z%), 439 (M^+^, 9.3), 302 (100), 151 (6.6).

**Diethyl [(5-Chloro-2-methylphenyl amino) (4-hydroxy-3-methoxy phenyl) methyl]phosphonate (16)**

This compound was synthesized after 90 min (64%. Mp = 152.4°C). 1H NMR (500 MHz, CDCl3): 7.01 (s, 1H, ArH), 6.95–6.98 (m, 2H, ArH), 6.91 (s, 1H, ArH), 6.65 (d, 1H, *J* = 6.1 Hz, ArH), 6.46 (s, 1H, ArH), 4.71 (s, 1H, CHP), 4.66 (s, 1H, NH), 4.09–4.19 (m, 2H, OCH2CH3), 3.96–4.01 (m, 1H, OCH2CH3), 3.89 (s, 3H, OCH3), 3.71–3.76 (m, 1H, OCH2CH3), 2.24 (s, 3H, CH3), 1.31 (t, 3H, *J* = 7.3 Hz, CH3), 1.29 (t, 3H, *J* = 7.3 Hz, CH3); 13C NMR (125 MHz, CDCl3): 147.45 (Ar-C), 146.17 (Ar-C), 145.09 (Ar-C), 132.82 (Ar-C), 131.38 (Ar-C), 127.24 (Ar-C), 121.65 (Ar-C), 121.13 (Ar-C), 118.21 (Ar-C), 115.12 (Ar-C), 111.79 (Ar-C), 110.63 (Ar-H), 63.91 (d, *J*_PC_ = 7.0 Hz, OCH2CH3), 63.73 (d, *J*_PC_ = 7.0 Hz CHP), 56.41 (OCH3), 56.13 (d, *J* = 151 Hz, CHP), 17.47 (CH3), 16.85 (d, *J*_PCH_ = 5.8 Hz, CH3), 16.68 (d, *J*_PCH_ = 5.75 Hz, CH3); MS: (m/z%), 413 (M+, 5.2), 276 (100), 137 (10.6).

**Diethyl [(5-Chloro-2-methylphenyl amino) (3,4-dimethoxy phenyl) methyl]phosphonate (17)**

This compound was synthesized after 90 min (70%. Mp = 135.2°C). ^1^H NMR (500 MHz, CDCl_3_): 7.02 (s, 1H, ArH), 6.97 (d, 2H, *J* = 8.1 Hz, ArH), 6.87 (d, 1H, *J* = 8.5 Hz, ArH), 6.64 (d, 1H, *J* = 8.1 Hz, ArH), 6.44 (s, 1H, ArH), 4.69 (d, 1H, *J* = 13.1 Hz, CHP), 4.68 (s, 1H, NH), 4.1–4.16 (m, 2H, OCH_2_CH_3_), 3.96–4.01 (m, 1H, OCH_2_CH_3_), 3.9 (s, 6H, OCH_3_), 3.71–3.75 (m, 1H, OCH_2_CH_3_), 2.24 (s, 3H, CH_3_), 1.32 (t, 3H, *J* = 7.3 Hz, CH_3_), 1.17 (t, 3H, *J* = 7.3 Hz, CH_3_); ^13^C NMR (125 MHz, CDCl_3_): 149.61 (Ar-C), 149.32 (Ar-C), 145.95 (Ar-C), 132.84 (Ar-C), 131.37 (Ar-C), 128.03 (Ar-C), 121.56 (Ar-C), 12.43 (Ar-C), 118.20 (Ar-C), 111.79 (Ar-C), 111.66 (Ar-C), 111.12 (Ar-H), 63.81 (d, *J*_PC_ = 6.8 Hz, OCH_2_CH_3_), 63.62 (d, *J*_PC_ = 7.0 Hz, OCH_2_CH_3_), 56.38 (OCH_3_), 56.27 (OCH_3_), 56.11 (d, *J* = 151 Hz, CHP), 17.49 (CH3), 16.87 (CH_3_), 16.72 (d, *J*_PCH_ = 5.8 Hz, CH_3_), 16.68 (d, *J*_PCH_ = 5.75 Hz, CH_3_); MS: (m/z%), 427 (M^+^, 6.6), 290 (100), 151 (9.6).

**Diethyl [(4-Chloro-2-nitrophenyl amino) (3,4-dimethoxyphenyl) methyl]phosphonate (18)**

This compound was synthesized after 120 min (65%. Mp = 161°C). ^1^H NMR (500 MHz, CDCl_3_): 8.87 (d, 1H, *J* = 10.5 Hz, ArH), 8.18 (s, 1H, ArH), 6.99 (s, 2H, ArH), 6.87 (d, 1H, *J* = 9.1 Hz, ArH), 6.69 (d, 1H, *J* = 9.1 Hz, ArH), 4.8 (d, 1H, *J*_CHPO_ = 23.1 Hz, CHP), 4.09–4.12 (m, 2H, OCH_2_CH_3_), 3.97–4.09 (m, 2H, OCH_2_CH_3_), 4.03–4.06 (m, 2H, OCH_2_CH_3_), 3.89 (s, 6H, OCH_3_), 1.23–1.32 (m, 6H, CH_3_); ^13^C NMR (125 MHz, CDCl_3_): 149.81 (Ar-C), 149.65 (Ar-C), 143.15 (Ar-C), 136.56 (Ar-C), 133.52 (Ar-C), 126.39 (Ar-C), 121.98 (Ar-C), 12030 (Ar-C), 116.66 (Ar-C), 111.72 (Ar-C), 110.88 (Ar-C), 64.16 (d, *J*_PC_ = 7.2 Hz, OCH_2_CH_3_), 63.97 (d, *J*_PC_ = 6.7 Hz, OCH_2_CH_3_), 56.44 (OCH_3_), 56.31 (OCH_3_), 55.77 (d, *J*_PC_ = 152 Hz CHP), 16.84 (d, *J*_PCH_ = 5.5 Hz, CH_3_), 16.76 (d, *J*_PCH_ = 5.6 Hz, CH_3_); MS: (m/z%), 458 (M^+^, 1.4), 321 (100), 165 (14).

**Diethyl [(4-Methoxy phenyl) (4-nitrophenyl amino) methyl]phosphonate (19)**

This compound was synthesized after 120 min (76%. Mp = 115°C). ^1^H NMR (500 MHz, CDCl_3_): 8.0 (d, 2H, *J* = 9.5 Hz, ArH), 7.42 (d, 2H, *J* = 8.7 Hz, ArH), 6.89 (d, 2H, *J* = 8.5 Hz, ArH), 6.64 (d, 2H, *J* = 9.5 Hz, ArH), 6.37 (t, 1H, *J* = 8.5 Hz, ArH), 4.81 (d, 1H,  *J*_CHPO_ = 23.5 Hz, CHP), 4.13–4.2 (m, 2H, OCH_2_CH_3_), 3.99–3.94 (m, 1H, OCH_2_CH_3_), 3.79 (s, 3H, OCH_3_), 3.67–3.72 (m, 1H, OCH_2_CH_3_), 1.30 (t, 3H, *J* = 7.3 Hz, CH_3_), 1.16 (t, 3H, *J* = 6.6 Hz, CH_3_); ^13^C NMR (125 MHz, CDCl_3_): 160.08 (Ar-C), 152.68 (Ar-C), 139.13 (Ar-C), 129.43 (Ar-C), 126.83 (Ar-C), 126.42 (Ar-C), 114.70 (Ar-C), 112.79 (Ar-C), 64.20 (d, *J*_PC_ = 7.0 Hz, OCH_2_CH_3_), 63.68 (d, *J*_PC_ = 7.0 Hz, OCH_2_CH_3_), 55.67 (OCH_3_), 55.29 (d, ^3^ *J*_PC_ = 7.5 Hz, CH_3_); MS: (m/z%), 394 (M^+^, 4), 257 (100), 211 (18), 121 (3.8).

**Diethyl [(4-Nitrophenyl)(phenyl amino)methyl]phosphonate (20)**

This compound was synthesized after 90 min (73%. Mp = 77°C). ^1^H NMR (500 MHz, CDCl_3_): 8.24 (d, 2H, *J* = 9.2 Hz, ArH), 7.70 (d, 2H, *J* = 8.5 Hz, ArH), 7.16 (t, 1H, *J* = 8.2 Hz, ArH), 6.78 (t, 1H, *J* = 7.5 Hz, ArH), 6.58 (d, 2H, *J* = 8.5 Hz, ArH), 4.89 (d, 1H, *J*_CHPO_ = 30.0 Hz, CHP), 4.87 (s. 1H, NH), 4.17–4.23 (m, 2H, OCH_2_CH_3_), 4.06–4.16 (m, 1H, OCH_2_CH_3_), 3.90–3.95 (m, 1H, OCH_2_CH_3_), 1.34 (t, 3H, *J* = 7.3 Hz, CH_3_), 1.23 (t, 3H, *J* = 7.3 Hz, CH_3_); ^13^C NMR (125 MHz, CDCl_3_): 148.07 (Ar-C), 146.06 (Ar-C), 144.49 (Ar-C), 129.79 (Ar-C), 129.06 (Ar-C), 124.19 (Ar-C), 119.55 (Ar-C), 114.24 (Ar-C), 64.16 (d, *J*_PC_ = 4.8 Hz, OCH_2_CH_3_), 63.9 (d, *J*_PC_ = 4.8 Hz, OCH_2_CH_3_), 56.48 (d, *J*_PC_ = 103 Hz, CHP), 16.84 (d, *J*_PCH3_ = 5.7 Hz, CH_3_), 16.67 (d, *J*_PCH3_ = 5.5 Hz, CH_3_); MS: (m/z%), 364 (M^+^, 7.9), 227 (100), 181 (23.8), 104 (3.6).

**Tetraethyl-1,4-phenylene bis((2-(1H-indolyl)ethylamino)methylene)diphosphonate (21)**

This compound was previously synthesized [29].

### 3.2. Modeling

The ligands were drawn in the Hyperchem 8. The geometry was optimized through the molecular dynamic method AMBER and semiempirical method PM3. The microtubule complexed with paclitaxol was obtained from Protein Data Bank (1JFF).

The Autodock software version 4.2 was used for the molecular docking process. The grids were constructed around the proteins. The Lamarckian Genetic Algorithm method was used for the global optimum binding position search. A number of 100 cycles of calculation were used in order to get a final binding position as accurate as possible. All the compounds as well as griseofulvin were docked into the active site of 1JFF. The complex of ligand-receptor was viewed by Accelry's Discovery Studio Visualizer. The docking procedure was run, and the maximum negative ΔG was calculated (Table 3).

### 3.3. Antifungal Assay

Microorganisms were obtained from the Mycology and Parasitology Department of the Shiraz University of Medical Sciences. Sabouraud dextrose agar (SDA), potato dextrose agar (PDA), and RPMI 1640 were used for agar dilution and microdilution methods. The clinical isolates of fungi including *M. canis, T. mentagrophytes, T. rubrum*,* E. floccosum*, and *C. albicans* were purified and subcultured on SC, SCC, and PDA media before testing. The stock solution of compounds was prepared in DMSO at a concentration of 200 mg/mL. The compounds were diluted in solid and broth media to obtain final concentration from 0.0625 to 2048 *μ*g/mL, using PDA and RPMI 1640 media. The inocula of the molds and yeast were prepared from 2–10 day mature colonies grown. Fluconazole and griseofulvin were used as positive and the solvents of the compounds as negative blanks.

## 4. Conclusion

*α*-Aminophosphonates are valuable compounds to be investigated as bioactive molecules and pharmacological agents. Recently, we have reported one-pot three-component synthesis starting from aldehydes, amines, and diethylphosphite using FeCl_3_ as a catalyst to formation of *α*-aminophosphonates [29]. In this study, synthesis of *α*-aminophosphonates using FeCl_3_ was compared with CuCl_2_. The results showed that FeCl_3_ is more efficient than CuCl_2_ as a catalyst for synthesis of *α*-aminophosphonates.

The biological assays show that only an indole containing bis-*α*-aminophosphonates has antifungal activity against M. canis. The docking results show that these compounds are candidate for cytotoxic activity studies.

## Figures and Tables

**Table 1:** Comparison of the effect of catalysts in preparation of *α*-aminophosphonate by the reaction of an aldehyde, aniline and diethylphosphite.

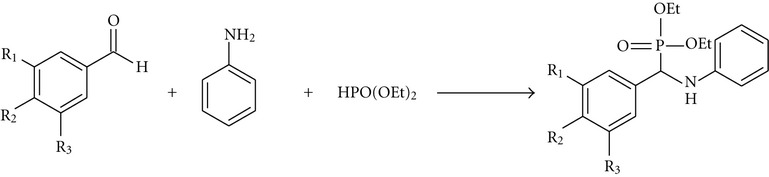				
Entry	Catalyst (0.1 mmol)	Solvent	Time (min)	Yield (%)

**1**	FeCl_3_	THF	30–120	73–84
**2**	CuCl_2_	THF	24	<5%

Compound **1**: R_1_  = OMe, R_2_  = OH, R_3_  = H; Compound **8**: R_1_  = Ome, R_2_  = Ome, R_3_  = H; Compound **11**: R_1_  = OMe, R_2_  = OMe, R_3_  = OMe; Compound **12**: R_1_ = H, R_2_  = OMe, R_3_ = H; Compound **14**: R_1_ = H, R_2_  = H, R_3_ = H; Compound **20**: R_1_ = H, R_2_  = NO_2_, R_3_ = H.

**Table 2:** FeCl_3_·THF solution catalyzed synthesis of bis(*α*-aminophosphonates) by using a three-component system.

Entry	Aldehyde	Amine	*α*-Aminophosphonate	Time (minutes)	Yield (%)
**1**	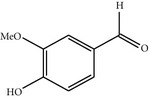	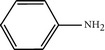	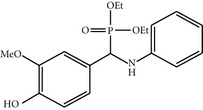	120	84
**2**	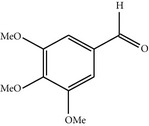	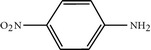	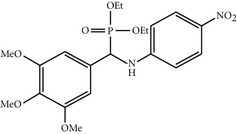	150	72
**3**	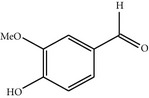	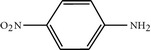	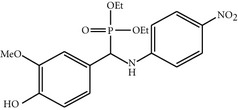	180	78
**4**	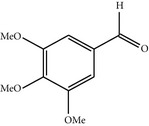	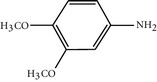	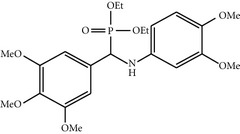	120	90
**5**	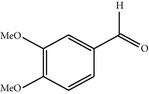	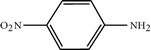	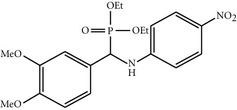	180	80
**6**	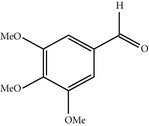	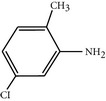	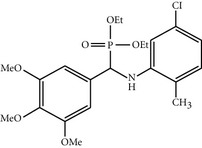	120	76
**7**	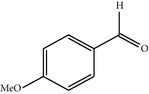	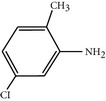	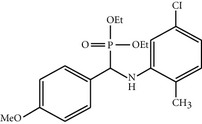	120	77
**8**	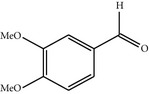	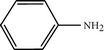	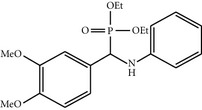	120	82
**9**	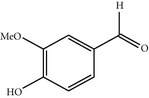	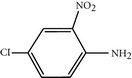	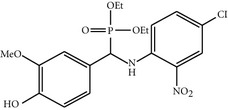	180	70
**10**	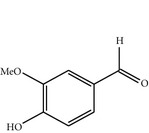	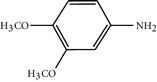	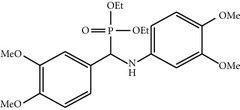	120	81
**11**	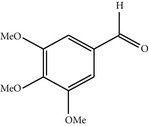	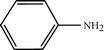	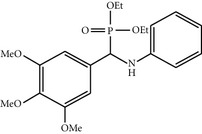	30	81
**12**	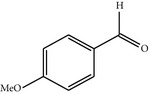	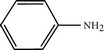	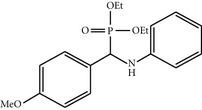	60	76
**13**	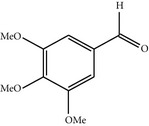	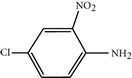	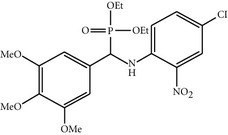	90	75
**14**	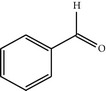	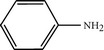	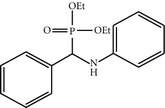	90	73
**15**	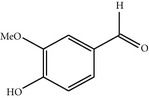	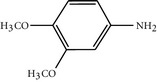	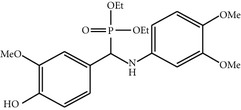	45	75
**16**	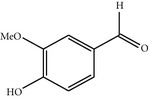	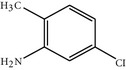	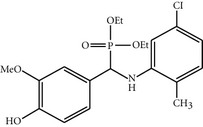	90	71
**17**	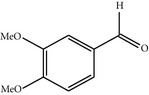	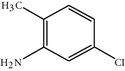	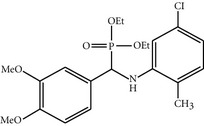	90	70
**18**	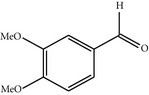	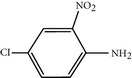	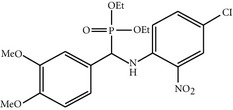	120	70
**19**	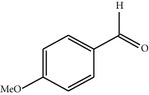	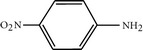	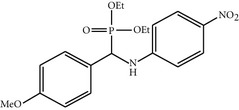	120	76
**20**	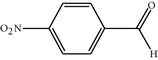	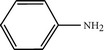	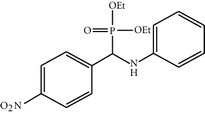	120	73
**21**	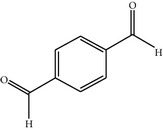	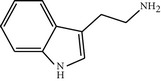	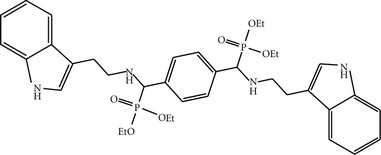	180	68

**Table 3:** Docking results of synthesized compounds into the active site of microtubule (1JFF).

Entry	ΔG (kcal/mol)	Entry	ΔG (kcal/mol)
**1**	−6.09	**12**	−5.55
**2**	−6.18	**13**	−5.88
**3**	−6.16	**14**	−5.79
**4**	−6.23	**15**	−5.49
**5**	−6.36	**16**	−6.22
**6**	−6.27	**17**	−6.34
**7**	−6.71	**18**	−6.10
**8**	−6.06	**19**	−6.61
**9**	−5.78	**20**	−6.74
**10**	−6.27	**21**	−7.4
**11**	−6.23	Griseofulvin	−6.76

**Table 4:** Antifungal activity of synthesized *α*-aminophosphonates.

Compound	*Candida albicans*	*Aspergillus flavus*	*Aspergillus fumigatus*	*Trichophyton mentagrophytes*	*Microsporum gypsum*	*Microsporum canis*	*Epidermophyton floccosum*
MIC *μ*g/mL							
**1**	G	G	G	2048	2048	G	G
**7**	G	G	G	2048	G	G	G
**9**	G	G	G	1024	G	G	G
**21**	G	G	G	G	G	0.5	G
Fluconazole	2	4	4	NT	NT	NT	NT
Griseofulvin	NT	NT	NT	0.5	8	0.6	1

G: Growth, NT: Not Tested.
